# A Low-Cost Fourier-Based Dual-Pump Mock Circulatory Loop for Arterial Pressure Waveform Reproduction

**DOI:** 10.3390/bioengineering13101104

**Published:** 2026-09-23

**Authors:** Victor K. Tsui, Kouhyar Tavakolian, Daniel Ewert

**Affiliations:** 1 Department of Biomedical Engineering, University of North Dakota, Grand Forks, ND 58202, USA; daniel.ewert@sdsmt.edu; 2 Department of Electrical and Computer Engineering, Old Dominion University, Norfolk, VA 23529, USA; ktavakol@odu.edu

**Keywords:** cardiovascular, mock circulatory loop, in vitro device testing, arterial pressure waveform, Fourier Transform, hemodynamic simulation, pulse-width modulation, dicrotic notch

## Abstract

Cardiovascular diseases are among the leading causes of death worldwide, creating a need for reliable hemodynamic testing platforms for cardiovascular device evaluation and therapeutic development. Mock circulatory loops (MCLs) provide effective in vitro platforms for reproducing physiological and pathological pressure conditions; however, many existing systems remain limited in programmability, waveform fidelity, standardization, and affordability. This study presents a cost-effective dual-pump hybrid MCL (hMCL) that independently regulates baseline and pulsatile pressure components using Fourier-based harmonic synthesis. Reference arterial pressure waveforms were decomposed into harmonic components, and their amplitudes, frequencies, and phase shifts were converted into pulse-width-modulation signals for dual-pump actuation. Validation using five physiological and virtual arterial pressure waveform datasets demonstrated MAP errors of ≤1.3%, RMSE values of 9.1–24.1 mmHg, and feature errors ≤ 2.8% for systolic, diastolic, and dicrotic notch pressures. Distribution analysis showed small median pressure differences and low variability across waveform characteristics, while repeated hMCL operation demonstrated pressure variations below 3 mmHg. These findings demonstrate that the proposed Fourier-based hMCL provides an accurate, reproducible, and cost-effective platform for arterial pressure waveform reproduction and early-stage cardiovascular device testing. The present validation is pressure-focused; flow waveform fidelity and pressure–flow interactions were not independently characterized.

## 1. Introduction

Cardiovascular disease (CVD) remains one of the leading causes of morbidity and mortality worldwide, accounting for approximately 19.8 million or 32% of global deaths annually, according to the World Health Organization [1]. In the United States, it accounts for a substantial proportion of annual deaths and healthcare expenditure [2]. Continued growth of the aging population, along with lifestyle-related risk factors, has intensified the demand for innovative cardiovascular therapies, medical devices, and diagnostic technologies. To bridge the gap between conceptual development and clinical implementation, reliable in vitro testing environments capable of reproducing realistic hemodynamic conditions are essential before advancing to animal studies or human trials.

Mock circulatory loops (MCLs) have emerged as indispensable platforms for cardiovascular device evaluation and physiological experimentation [3]. An MCL enables controlled simulation of pulsatile pressure and flow, replicating the mechanical behavior of the heart, vascular compliance, peripheral resistance [4], and pathological conditions [5]. These systems are widely applied in heart valve development, ventricular assist device testing, endovascular implant evaluation, medical training, and hemodynamics research [6]. Despite their broad utility, most existing MCLs remain custom-built, mechanically complex, and costly. As a result, reproducibility across laboratories is limited, and waveform fidelity often varies significantly between systems. The lack of standardization further limits scalability, accessibility, and the meaningful comparison of experimental results across studies [7]. Recent efforts have begun integrating computational control systems and real-time feedback mechanisms to improve flexibility and precision in pressure waveform reproduction. Nevertheless, a universally adaptable, programmable, and affordable MCL architecture has yet to be realized [8]. Few low-cost MCL platforms enable programmable arterial waveform reconstruction while accurately preserving clinically relevant pressure features, including systolic, diastolic, and dicrotic notch pressures.

In this study, we present a programmable hybrid MCL (hMCL) that employs a dual-pump pulse-width-modulation (PWM) control strategy based on Fourier harmonic synthesis of arterial pressure waveforms. We hypothesized that a reduced-order Fourier representation, combined with independent baseline and pulsatile pump control, could reproduce key arterial pressure features with sufficient accuracy for educational, research, and early-stage device-testing applications. Reference arterial waveforms are decomposed into harmonic components using the Fast Fourier Transform (FFT), and the resulting harmonic amplitudes, frequencies, and relative phase shifts are used to reconstruct physiological arterial pressure waveforms through coordinated control of two independently driven centrifugal pumps. This approach enables programmable generation of physiologically representative arterial waveforms and accurate reproduction of key hemodynamic features, including systolic, diastolic, and dicrotic notch pressures, within a simple and cost-effective architecture.

The primary contribution of this work is the development of a low-cost hardware-based programmable hMCL that combines Fourier harmonic synthesis with independent regulation of baseline and pulsatile pressures using a dual-pump architecture. Unlike conventional MCLs that primarily rely on mechanical adjustment of compliance and resistance elements or direct waveform playback to reproduce arterial pressure waveforms, the proposed system separates baseline pressure generation from pulsatile pressure waveform synthesis, enabling programmable reconstruction of physiological arterial pressure waveforms using a limited number of harmonics while maintaining a hardware component cost below US$200 as of this publication date. System performance was validated using five publicly available physiological and virtual arterial waveform datasets and evaluated through mean arterial pressure (MAP) error, root mean square error (RMSE), feature-based pressure error analysis, descriptive statistical analysis of pressure differences, and repeatability testing [9,10,11,12,13]. The resulting hMCL provides an economical, accessible, and programmable platform for early-stage cardiovascular device testing, hemodynamic research, and biomedical engineering education.

## 2. Materials and Methods

### 2.1. Fast Fourier Transform Decomposition

In this study, each arterial pressure waveform x(t) is decomposed into a series of sinusoidal components using the Fourier series representation. A periodic waveform can be expressed as Equation (1):

(1) $$x(t)=a_{0}+\sum_{n=1}^{\infty }\left[a_{n}cos(n\omega_{0}t)+b_{n}sin(n\omega_{0}t)\right]$$

where

- $\omega_{0}=\frac{2\pi }{T}$ is the fundamental angular frequency;
- T is the waveform period;
- n is the harmonic number;
- $a_{0}$ is the DC (direct current) component or constant term as baseline;
- $a_{n}$ and $b_{n}$ are Fourier series coefficients representing the amplitude and phase of each harmonic.

The amplitude and phase of each harmonic are given by Equation (2):

(2) $$A_{n}=\sqrt{a_{n}^{2}+b_{n}^{2}},\;and{\;\phi }_{n}=arctan\left(\frac{b_{n}}{a_{n}}\right)$$

### 2.2. Fourier-Based Harmonic Synthesis

To generate a simplified yet physiologically representative arterial waveform, only the first three harmonics (n=1,2,3) were used to reconstruct an approximation of the original arterial pressure waveform Equation (3):

(3) $$x_{\left(n=1:3\right)}\left(t\right)=a_{0}+\sum_{n=1}^{3}A_{n}\cos\left(n\omega_{0}t+\phi_{n}\right)$$

The first three harmonics (H1–H3) capture the dominant frequency content of arterial pressure waveforms, including the systolic upstroke, dicrotic notch, and diastolic decay. To determine the number of harmonics required for waveform reconstruction, FFT analysis was performed on the five reference arterial waveform datasets. As summarized in Table 1, the normalized amplitudes and corresponding phase shifts were calculated for the baseline (DC) component and the first through fourth harmonics. Across all datasets, the first harmonic exhibited the largest oscillatory amplitude, followed by progressively smaller second and third harmonics. In contrast, the fourth harmonic contributed only 0.5–1.9% of the normalized full-scale waveform amplitude (0–100%). Because the spectral power of each harmonic is proportional to the square of its amplitude, the cumulative spectral power contained in H1–H3 exceeded 98.8% for all five datasets, whereas the fourth harmonic (H4) contributed only 0.1–1.2% of the total power. These results indicate that H1–H3 capture nearly all the physiologically relevant oscillatory content of the reference arterial pressure waveforms.

The RMSE comparison in Table 1 further supports the selection of the first three harmonics for waveform reconstruction. Adding the fourth harmonic reduced the RMSE by only 0.09–0.30 mmHg across the five datasets. Although H4 provided a measurable improvement, its incremental contribution to waveform reconstruction was small relative to the additional high-frequency component introduced. Therefore, waveform synthesis was limited to H1–H3, which provided a practical balance between waveform reconstruction accuracy, control complexity, and the dynamic limitations of the centrifugal pumps. With fundamental frequency f (Hz) and $\omega_{0}=2\pi f$, the waveform reconstructed using the first three harmonics is expressed as:

(4) $$x_{\left(n=1:3\right)}(t)=a_{0}+\sum_{n=1}^{3}A_{n}cos(2\pi nft+\phi_{n})$$

For waveform reconstruction, the fundamental harmonic was selected as the phase reference, and its phase was set to zero ($\phi_{1}=0)$. The reconstructed waveform can therefore be written as Equation (5):

(5) $$x_{\left(n=1:3\right)}(t)=a_{0}+A_{1}cos(2\pi ft)+A_{2}cos(4\pi ft+\phi_{2})+A_{3}cos(6\pi ft+\phi_{3})$$

Under this phase-reference convention, the phases of the second and third harmonics are expressed relative to the fundamental harmonic, preserving their phase relationships with the fundamental component. This approach enables reconstruction of the principal morphological characteristics of the arterial pressure waveform, including systolic, diastolic, and dicrotic notch pressures.

### 2.3. Pulse-Width-Modulation Conversion

The synthesized Fourier waveform was converted into a pulse-width-modulation (PWM) signal to control pump speed [14].

The baseline component was applied to the baseline pump, while the synthesized pulsatile component was applied to the waveform-shaping pump. PWM duty cycle was used to regulate the rotational speed of each centrifugal pump and, consequently, the pressure generated within the hMCL. An L298N motor driver from Amazon interfaced the 5 V microcontroller control signals with the 6–12 V motor power supply.

The resulting PWM signals were transmitted from the Arduino microcontroller to the motor driver for dual-pump actuation. The detailed PWM scaling, feedback regulation, and pressure-control architecture are described in the System Architecture Section. The waveform decomposition and reconstruction process is illustrated in Figure 1.

A dual-pump architecture was selected to reduce the reliance on a single large high-power pump, which typically introduces higher mechanical noise, higher activation power requirements, and increased system cost. Instead, the proposed system utilizes two smaller low-power 10-Watt pumps operating cooperatively to generate baseline and pulsatile flow components independently. This configuration improves controllability of low-amplitude waveform features while reducing mechanical vibration and startup power demand. Separating steady-state pressure generation from pulsatile waveform-shaping also enables more stable PWM control and enhances flexibility in reproducing physiological pressure profiles.

### 2.4. System Architecture

MCLs are generally classified into three categories: mechanical, computational, and hybrid systems [15,16,17,18,19]. Mechanical MCLs use physical components, such as pumps, compliance chambers, reservoirs, and valves, to reproduce physiological pressure and flow conditions for in vitro cardiovascular device testing. Computational MCLs simulate the cardiovascular system numerically and provide highly programmable hemodynamic modeling but cannot physically evaluate cardiovascular devices. The hMCLs combine a physical fluid circuit with computational control, enabling programmable waveform generation while maintaining compatibility with experimental device testing. The system developed in this study belongs to the hMCL category, integrating a programmable Fourier-based dual-pump control strategy with a simplified hydraulic circulation loop to provide a flexible and low-cost hardware platform for cardiovascular waveform simulation.

The hMCL implementation consists of a simplified systemic circulation consisting of a dual-pump outlet, arterial compliance chamber, peripheral resistance, and venous reservoir, representing the essential components of the systemic circulation. The overall hemodynamic behavior is consistent with a first-order Windkessel arterial model, which describes the relationship among arterial pressure, flow, arterial compliance, and peripheral resistance [20]. The governing equation of the first-order Windkessel model is expressed as Equation (6):

(6) $$C\frac{dP(t)}{dt}+\frac{P(t)}{R}=Q(t)$$

where

- C is arterial compliance;
- R is peripheral resistance;
- $P\left(t\right)$ is arterial pressure;
- $Q\left(t\right)$ is the pump-generated flow.

A first-order two-element Windkessel model was used to represent the simplified systemic arterial load in the present study. This model was selected to provide a computationally simple representation of arterial compliance and peripheral resistance consistent with the pressure-focused objective of the prototype. Although a three-element Windkessel model incorporating characteristic arterial impedance provides a more comprehensive representation of arterial pressure–flow dynamics, its implementation was beyond the scope of the present pressure-only proof-of-concept system. Extension to a three-element model with direct flow measurement and pressure–flow validation represents an important direction for future development.

The control architecture and data acquisition framework were developed using MATLAB/Simulink R2026a because of its numerical computing capabilities and native support for real-time hardware interfacing with Arduino microcontrollers. The overall control architecture of the proposed hMCL is illustrated in Figure 2.

Reference arterial pressure waveforms were decomposed into harmonic components using the FFT. The DC component was assigned to the baseline pump, whereas the H1–H3 components were used to modulate the pulsatile pump. The DC and pulsatile harmonic components were normalized to signal factors ranging from 0 to 1. These signal factors were subsequently multiplied by their corresponding PWM commands generated in MATLAB/Simulink and transmitted through an Arduino UNO microcontroller to the motor driver, as described in Equation (7).

(7) $${PWM}_{final}(t)=SF(t)\times {PWM}_{command}(t)$$

where

- ${PWM}_{final}$ is the resulting PWM signal, ranging from 0 to 255, applied by the motor driver controller to the corresponding pump;
- SF is the normalized signal factor, ranging from 0 to 1, which is a constant value for baseline pump control and a time-varying waveform for pulsatile pump control;
- ${PWM}_{command}\left(t\right)$ is the adjusted PWM command generated by the PI controller.

Pressure signals from the two sensors were sampled at 100 Hz by the Arduino microcontroller and transmitted to MATLAB/Simulink for real-time feedback control. The arterial pressure waveform was buffered over 1.5 s to cover one cardiac cycle, and the maximum and minimum pressures were extracted as systolic and diastolic values, respectively. The measured systolic and diastolic pressures were compared with their corresponding target setpoints, and two proportional–integral (PI) controllers independently adjusted the PWM commands of the pulsatile and baseline pumps, respectively. This dual-loop control architecture enabled independent regulation of systolic and diastolic pressures while maintaining the target waveform during closed-loop operation. A schematic of the dual-pump hMCL system is presented in Figure 3.

### 2.5. Experimental Setup and Calibration

The dual-pump hMCL was constructed using two 10-Watt centrifugal pumps. Centrifugal pumps were selected due to their low operational noise and reduced minimum activation power compared to large single-pump systems, particularly positive-displacement pumps. The systemic circuit was assembled using elastic silicone tubing to mimic arterial compliance and vessel elasticity. A 2 L urine bag served as the fluid reservoir and blood analog volume source, while an inline flow regulator was used to adjust and maintain overall peripheral resistance. System operation and data acquisition were controlled by an Arduino UNO microcontroller, with the physical setup illustrated in Figure 4 [21]. The materials and associated system costs are summarized in Table 2.

Following construction of the hMCL, both pressure sensors were calibrated offline against an external digital reference manometer connected in parallel with a 60 mL syringe used to generate controlled static pressure inputs. A two-point calibration was selected because the pressure transducer was treated as having an approximately linear voltage–pressure response over the operating range. Two calibration points are sufficient to determine the gain and offset of a linear calibration relationship. The pressure transducer has a full-scale (FS) range of approximately 517 mmHg and a specified accuracy of ±1% FS, corresponding to approximately ±5.2 mmHg over the full measurement range. Calibration was performed at 0 mmHg and a 300 mmHg reference pressure using an external reference pressure measurement. The 300 mmHg calibration point was selected within the upper portion of the sensor’s measurement range to provide a representative calibration span without relying on the full-scale endpoint. This approach was considered adequate for the pressure range and accuracy requirements of the present study. The resulting linear calibration relationship was expressed as Equation (8):

(8) P=mD+c

where

- P is the calibrated pressure (mmHg);
- D is the corresponding Arduino digital sensor reading;
- m is the gain (scaling coefficient);
- c is the offset coefficient.

These calibration coefficients were subsequently incorporated into the Simulink framework to ensure accurate and consistent pressure measurements during system operation.

Flow rate was measured using an in-line impeller-type flow meter and calibrated using a gravimetric collection method. During the experiments, the flow regulator was adjusted primarily to obtain the desired pressure range and, where necessary, to achieve the required systolic pressure slope. Consequently, the flow rate was monitored to ensure stable circulation within the hydraulic loop but was not used as a validation metric. Because the primary objective of the present study was pressure waveform reproduction, the selected flow range should be considered a practical operating condition rather than a quantitative representation of a specific artery or physiological state. Accordingly, flow rate and the non-Newtonian rheological properties of blood were not included as evaluation parameters. The proposed dual-pump control architecture is designed to independently regulate baseline and pulsatile pressures, enabling programmable generation of physiological arterial pressure waveforms and providing flexibility to accommodate different arterial loading conditions. However, because flow waveform fidelity and cardiovascular pressure–flow interactions were not independently validated in this study, the current system should be regarded as a pressure waveform reproduction platform rather than a complete physiological pressure–flow simulator.

### 2.6. System Validation

The proposed hMCL was validated using five reference arterial pressure waveform datasets, including (1) physiological radial arterial waveforms from the PhysioNet CHARIS database [22], (2) physiological radial arterial waveforms from the MIMIC III database [23], (3) virtual radial arterial waveforms from the FDA MCL database [24], and (4) virtual brachial and (5) femoral arterial waveforms from King’s College London datasets [25]. The harmonic parameters extracted from each reference waveform were used to program the hMCL and generate the corresponding target arterial pressure waveform.

Waveform fidelity was evaluated using five complementary approaches: (1) MAP error to assess clinically relevant pressure reproduction; (2) RMSE to quantify overall waveform similarity; (3) feature-based error analysis of systolic, diastolic, and dicrotic notch pressures to evaluate preservation of key hemodynamic characteristics; (4) descriptive statistical analysis of pressure differences using boxplots to summarize the central tendency and variability of waveform feature errors; and (5) repeatability analysis across five independent operating sessions to evaluate the consistency and reliability of pressure waveform generation.

For descriptive statistical analysis, the hMCL was allowed to reach steady-state operation before data acquisition for each reference dataset. Within-session repeatability was evaluated using ten consecutive stabilized cardiac cycles, representing a single steady-state operating interval after transient effects had dissipated. These cycles provided sufficient samples to characterize the variability of waveform features while maintaining consistent operating conditions. Systolic pressure, diastolic pressure, dicrotic notch pressure, and mean arterial pressure (MAP) were extracted from each cardiac cycle and compared with the corresponding reference waveform features. For each of the five reference datasets, the mean error, standard deviation (SD), median, interquartile range (IQR), 95% confidence interval (CI), and 95% limits of agreement (LoA) of the mean pressure differences were calculated. These descriptive statistics characterized the distribution and within-session variability of the pressure differences between the hMCL-generated and reference waveforms. Because these measurements were obtained from consecutive cardiac cycles within a single operating session, the descriptive statistics characterize within-session repeatability rather than between-session reproducibility.

To evaluate the repeatability of the proposed hMCL, the FDA MCL dataset (3) was selected as the reference waveform. The hMCL was programmed to reproduce the target waveform and operated for five independent one-minute trials under identical operating conditions. To eliminate startup transients, the initial 20 s of each trial were excluded, and only the stabilized waveform segment was analyzed. Systolic pressure, diastolic pressure, and dicrotic notch pressure were extracted from the stabilized waveforms of each trial, and the corresponding mean values and standard deviations were calculated to evaluate the waveform generation consistency and pressure regulation repeatability.

Long-term operational stability of the proposed hMCL was evaluated during a continuous 4 h experiment under operating conditions equivalent to those used in the repeatability evaluation. The system was operated continuously while generating the same reference arterial pressure waveform. Measurements were evaluated at the beginning of the experiment (0 h) and after 1, 2, 3, and 4 h of continuous operation. At each point, a 40 s stabilized recording window was used for analysis, consistent with the data window used for the repeatability evaluation. Systolic pressure, diastolic pressure, and dicrotic notch pressure were identified from the recorded cardiac cycles within each 40 s window, and the mean and standard deviation (SD) were calculated for each pressure feature. The coefficient of variation (CV) across the five time-point mean values was calculated to quantify temporal variability over the 4 h operating period. This experiment was performed to evaluate long-term pressure stability and identify potential temporal drift during continuous operation.

## 3. Results

The proposed hMCL was operated under Simulink control using the five reference datasets described in Section 2.6. For each experiment, pressure signals were continuously acquired for one minute through the Simulink–Arduino interface and exported to MATLAB for subsequent waveform analysis. Figure 5 presents a representative screen capture of the real-time system operation, including the generated arterial pressure output, return pressure, and reconstructed input waveform used for pump control. After a brief startup transient, the system reached a stable periodic operating state and maintained repeatable pressure waveforms throughout the recording period, providing consistent data for subsequent quantitative waveform validation.

### 3.1. Waveform Error Analysis

Representative waveform analysis and feature extraction results for the five reference datasets are shown in Figure 6. For each dataset, the reference and hMCL-generated waveforms were analyzed using the same fiducial features, including baseline pressure, systolic pressure, anacrotic slope, dicrotic notch pressure, end-diastolic pressure, and dicrotic slope. The overlay comparisons demonstrate close agreement in the principal waveform morphology, with localized differences primarily observed around the systolic upstroke and dicrotic transition. Quantitative RMSE and feature-error results are summarized in Table 3.

Because the hMCL waveform exhibited a reproducible temporal displacement relative to the reference waveform, both unshifted and time-aligned RMSE values were reported. The unshifted RMSE reflects the combined effects of amplitude, morphology, and temporal mismatch, whereas the time-aligned RMSE provides a morphology-focused assessment after compensating for the observed temporal displacement. This displacement may result from multiple factors, including pump dynamic response, hydraulic wave propagation, system compliance, sensor response, data acquisition latency, and the FFT-based waveform generation process.

Temporal alignment was performed by systematically shifting the hMCL waveform along the time axis and calculating the RMSE relative to the reference waveform at each shift. The time shift producing the minimum RMSE was selected as the optimal alignment. Figure 7 shows the reference and time-shifted hMCL waveforms for the five representative datasets, demonstrating improved temporal synchronization after alignment. The corresponding RMSE values before and after time shifting are summarized in Table 3.

### 3.2. Descriptive Statistical Analysis

The grouped boxplots (Figure 8) demonstrate that the pressure differences between the hMCL-generated and reference waveforms were generally centered near zero, indicating minimal systematic bias. A larger positive bias was observed for systolic pressure in the London—Brachial dataset, with a mean difference of 3.81 ± 1.08 mmHg. The IQRs were generally narrow for systolic, diastolic, and MAP measurements, indicating low variability and consistent waveform reproduction. Greater variability was observed for the dicrotic notch, particularly for the CHARIS dataset, which showed a mean difference of −4.18 ± 2.25 mmHg and an IQR of 3.05 mmHg. This greater variability consists of the increased sensitivity of the dicrotic notch to waveform morphology, timing, and the dynamic response characteristics of the hydraulic system. Individual outliers were retained because they represent observed waveform variability rather than measurement errors. As summarized in Table 4, the mean difference ± SD, median, IQR, 95% CI, and 95% LoA were calculated for systolic pressure, diastolic pressure, dicrotic notch pressure, and MAP across all five reference datasets. Across datasets, systolic, diastolic, and MAP mean differences were generally small, with the overall mean differences of 0.40, 0.43, and 0.38 mmHg, respectively. The overall dicrotic notch mean difference was −0.92 mmHg. The overall SDs were 2.27 mmHg for systolic, 1.83 mmHg for diastolic, 2.98 mmHg for dicrotic notch, and 1.05 mmHg for MAP. The 95% LoA for systolic, diastolic, and MAP were generally narrow, although some individual datasets exceeded ±5 mmHg; the dicrotic notch exhibited wider limits of agreement, ranging from −8.59 to 0.23 mmHg for CHARIS. These results indicate low systematic bias and generally low variability in the reproduction of arterial pressure characteristics across the five reference datasets.

### 3.3. Repeatability Evaluation

The repeatability of the proposed hMCL was assessed over five independent one-minute trials under identical operating conditions. As summarized in Table 5, the standard deviations of systolic pressure, diastolic pressure, and dicrotic notch pressure remained below 3 mmHg and coefficients of variation below 3% across five independent trials, indicating high repeatability and stable pressure regulation. These findings demonstrate that the proposed hMCL can reproducibly generate physiologically representative arterial pressure waveforms during repeated operation.

### 3.4. Long-Term Stability

The long-term stability of the hMCL was evaluated during 4 h of continuous operation. As shown in Figure 9, the generated arterial pressure remained stable throughout the experiment, with no observable progressive drift in systolic pressure, diastolic pressure, or dicrotic notch pressure. The mean diastolic pressure ranged from 91.83 to 93.84 mmHg, with SDs of 0.93 to 1.62 mmHg, while mean systolic pressure ranged from 147.04 to 149.42 mmHg, with SDs of 1.16 to 1.28 mmHg. Dicrotic notch pressure ranged from 103.46 to 106.09 mmHg, with SDs of 1.18 to 1.95 mmHg. The corresponding temporal coefficients of variation across the five measurement points were 0.85%, 1.26%, and 1.46% for systolic, diastolic, and dicrotic notch pressures, respectively. These low coefficients of variation and narrow pressure ranges demonstrate stable waveform generation over the 4 h test period.

## 4. Discussion and Limitations

### 4.1. Principal Findings

The proposed Fourier-based dual-pump hMCL demonstrated that physiologically representative arterial pressure waveforms can be reproduced using a simple, programmable, and cost-effective platform capable of independently regulating systolic pulsatility and diastolic pressure. This capability is particularly advantageous for early-stage cardiovascular device testing and biomedical engineering education, where flexible and customizable hemodynamic conditions are desirable. Compared with conventional MCL systems, the proposed architecture, implemented using an Arduino-based control framework, provides enhanced waveform programmability and successfully reproduces key arterial waveform characteristics, including systolic pressure, diastolic pressure, and the dicrotic notch. Waveform error analysis demonstrated a high level of agreement between hMCL-generated and reference waveforms. Validation based on clinically relevant hemodynamic features, including systolic pressure, diastolic pressure, dicrotic notch pressure, and MAP, showed errors of less than 3%, indicating that the Fourier-based waveform reconstruction approach preserved the essential characteristics of physiological arterial pressure waveforms. Furthermore, repeatability testing further demonstrated good system stability and reproducibility across repeated measurements. The low variability observed among repeated trials indicates that the control strategy and calibration procedure provided consistent pressure generation and waveform generation performance. This repeatable behavior is important for cardiovascular device evaluation, where reliable testing conditions are required to ensure meaningful comparisons between experimental trials.

The RMSE values ranged from 9.1 to 24.1 mmHg across the five evaluated datasets, corresponding to approximately 6–16% of the full-scale pressure range represented by the waveforms. Although these values appear relatively large compared with the feature-based errors, RMSE reflects point-by-point differences across the entire cardiac cycle and is particularly sensitive to temporal shifts during rapid systolic upstroke and dicrotic notch formation. To assess the effect of this temporal displacement, a time-shifting approach was applied to minimize RMSE between the hMCL-generated and reference waveforms. Following temporal alignment, RMSE decreased to 4.9–13.7 mmHg, corresponding to approximately 3–9% of the full-scale pressure range. These findings indicate that a substantial portion of the unshifted RMSE may be associated with reproducible temporal displacement between the reference and hMCL waveforms rather than differences in waveform morphology alone. After temporal alignment, the residual differences were primarily observed during the anacrotic upstroke, dicrotic notch transition, and diastolic decay phases, suggesting that the proposed hMCL reproduced the overall waveform morphology with good fidelity. The time-aligned RMSE should therefore be interpreted as a morphology-focused metric rather than as the unmodified real-time waveform error.

The descriptive statistical analysis demonstrated consistently small differences in systolic pressure, diastolic pressure, and mean arterial pressure (MAP), indicating that the proposed Fourier-based dual-pump control strategy accurately reproduced the principal hemodynamic characteristics of the reference waveforms. Across the five reference datasets, mean differences for these pressure features were below 1 mmHg, with standard deviations below 3 mmHg, demonstrating low variability and strong agreement. Dicrotic notch pressure showed greater variability, likely because the notch region is more sensitive to small changes in pressure decay, wave-reflection timing, and transient hydraulic response. Nevertheless, the dicrotic notch remained consistently identifiable in all generated waveforms, indicating preservation of the principal waveform morphology.

The repeatability experiments further demonstrated consistent pressure generation across five independent trials, with standard deviations below 3 mmHg and coefficients of variation below 3% for systolic, diastolic, and dicrotic notch pressures. In addition, the 4 h continuous operation experiment showed no apparent progressive drift, with temporal coefficients of variation of 0.85%, 1.26%, and 1.46% for systolic, diastolic, and dicrotic notch pressures, respectively. Together, these results demonstrate both short-term repeatability and sustained pressure stability of the hMCL. Importantly, this performance was achieved using commercially available components with a hardware cost below US$200, supporting the potential of the proposed system as a low-cost platform for cardiovascular research and biomedical engineering applications.

### 4.2. Dicrotic Notch Reproduction

The Fourier-based harmonic decomposition and reconstruction approach enables the conversion of target waveform harmonics into PWM control signals for dual-pump actuation. This reconstruction method facilitates the generation of key physiological features, including the dicrotic notch (incisura). The dicrotic notch is a critical feature of the arterial pressure waveform, reflecting aortic valve closure and transient flow reversal in the proximal aorta. Its morphology and timing are strongly influenced by arterial wall elasticity; compliant arteries promote wave reflection and energy storage, resulting in a well-defined notch, whereas increased stiffness attenuates or shifts this feature. Clinically, the dicrotic notch provides valuable insight into cardiovascular function, particularly in pathological conditions such as severe aortic stenosis, where altered valve dynamics and reduced stroke volume can produce a diminished or delayed incisura. Therefore, accurate representation of the dicrotic notch is essential for reliable waveform analysis and generation [26]. Moreover, this strategy enables flexible generation of both physiological and pathological arterial pressure waveforms without requiring extensive mechanical modification of the circulatory loop. As a result, the proposed hMCL platform offers a practical and accessible solution for cardiovascular research, early-stage device evaluation, and educational training.

### 4.3. Comparison with Existing MCL Systems

A comparative summary highlighting the differences between the proposed hMCL and conventional MCL designs is presented in Table 6 [3,4,6,20,27]. The simplified architecture, low component cost, and programmable waveform generation capabilities represent key advantages of the proposed system. Unlike many conventional MCLs that rely on direct waveform playback or extensive mechanical tuning of compliance and resistance elements, the proposed hMCL reconstructs arterial pressure waveforms using Fourier-based harmonic synthesis and independently regulates baseline and pulsatile pressure components through a dual-pump control architecture [28]. This approach enables flexible waveform customization while maintaining accurate reproduction of key hemodynamic features, including systolic pressure, diastolic pressure, and the dicrotic notch. The ability to reproduce physiologically representative dicrotic notch morphology further enhances waveform realism and provides an additional advantage over conventional MCL implementations that rely primarily on mechanical tuning or direct waveform playback.

From a translational perspective, the proposed hMCL is intended primarily as a low-cost preclinical and benchtop platform rather than a clinical diagnostic system. Its programmable pressure waveform generation could support early-stage cardiovascular device evaluation by enabling controlled testing under different physiological and pathological arterial pressure conditions. The independent regulation of baseline and pulsatile pressure may also facilitate investigation of device responses to changes in systolic and diastolic loading conditions. Such a platform could reduce the cost and complexity of preliminary device development and provide an accessible test environment for biomedical engineering research and education. However, clinical translation would require additional validation against established MCL systems and clinically relevant hemodynamic conditions, including direct flow measurement, pressure–flow characterization, long-term stability, and assessment of physiological parameter accuracy. Therefore, the current system should be considered a proof-of-concept pressure-based platform for early-stage cardiovascular research rather than a clinically validated testing device.

### 4.4. Limitations

Several limitations should be acknowledged. First, the present study focused exclusively on arterial pressure waveform reproduction. Although cardiac output was estimated from the pump operating conditions, the fidelity of the flow waveform was not independently validated against reference datasets. Consequently, the current prototype should be considered a pressure-only MCL and is not intended for applications requiring validated pressure–flow characterization. Future work will incorporate direct flow waveform measurements and closed-loop control of both pressure and cardiac output. Second, water was used as the blood analog fluid. Because the viscosity of blood is approximately three times greater than that of water, the experimental conditions may not accurately reflect physiological hemodynamics. These differences in fluid properties may influence vascular resistance, compliance behavior, and pressure–flow relationships. Third, waveform fidelity was constrained by the transient response and finite bandwidth of the centrifugal pumps and hydraulic circuit. Although the proposed hMCL accurately reproduced the principal arterial pressure waveform characteristics, rapid systolic upstroke, dicrotic notch formation, and pressure decay remained sensitive to the dynamic response limits of the system. Future improvements in pump actuation speed, closed-loop control performance, and hydraulic compliance tuning may further enhance waveform fidelity.

Finally, the performance of the current system is constrained by the output capability of the centrifugal pumps. The maximum achievable systolic upstroke slope was approximately 350 mmHg/s, while the maximum dicrotic decay slope was approximately −250 mmHg/s during PWM characterization. Consequently, rapid systolic acceleration and steep dicrotic transitions observed in certain physiological waveforms may not be fully reproduced. In addition, the operating pressure range of approximately 50–220 mmHg limits simulation of extreme hypotensive and hypertensive conditions. Despite these limitations, the proposed hMCL demonstrated sufficient performance for waveform visualization, biomedical engineering education, early-stage cardiovascular device testing, and hemodynamic research applications.

## 5. Conclusions and Future Work

This study presented a low-cost hardware-based programmable dual-pump hMCL capable of reproducing physiologically representative arterial pressure waveforms using a Fourier-based harmonic synthesis approach. By combining two independently controlled centrifugal pumps with Arduino-based PWM closed-loop control, the proposed system provides a simple, flexible, and economical platform for physiological waveform generation.

Validation against five physiological and virtual reference waveform datasets demonstrated that MAP errors remained ≤1.3%, feature errors were ≤2.8%, and overall mean differences were below 1 mmHg for systolic, diastolic, dicrotic notch, and MAP measurements, supporting the reproducibility of the pressure waveform generation approach. These results demonstrate that the proposed Fourier-based hMCL provides an accurate, reproducible, and cost-effective platform for early-stage cardiovascular device testing under controlled arterial pressure conditions.

Several opportunities exist for future system enhancement. During this study, the original Arduino UNO R3 controller was upgraded to the Arduino UNO R4 WiFi platform, providing increased memory capacity for advanced control algorithms and improved 14-bit analog-to-digital conversion for pressure sensor acquisition. Future work will focus on improving pump transient response, increasing pressure slew rate during systolic upstroke, optimizing closed-loop control, and refining hydraulic compliance to further enhance waveform fidelity.

Additional improvements may include integration of wireless communication through Bluetooth or WiFi for remote monitoring, distributed laboratory instruction, and educational demonstrations. Future work will also incorporate higher-precision, fast-response flow sensors and closed-loop flow regulation to improve flow measurement and enable validation of physiological flow reproduction and pressure–flow relationships. Blood analog fluids with properties closer to those of human blood should be used in place of water to better replicate physiological hemodynamics and improve pressure and flow validation. Advanced closed-loop control methods may further improve waveform fidelity during rapid systolic upstroke and dicrotic notch formation. The waveform library will also be expanded to include a broader range of physiological and pathological cardiovascular conditions, enabling simulation of diverse arterial pressure waveform morphologies and hemodynamic states. Collectively, the low cost, waveform programmability, and reproducible pressure waveform generation of the proposed hMCL may facilitate broader adoption for cardiovascular research, early-stage medical device evaluation, biomedical engineering education, and resource-constrained laboratories.

## 6. Patents

The technology described in this work is the subject of a pending patent application related to the programmable dual-pump mock circulatory loop and arterial waveform generation methodology.

## Figures and Tables

**Figure 1:**
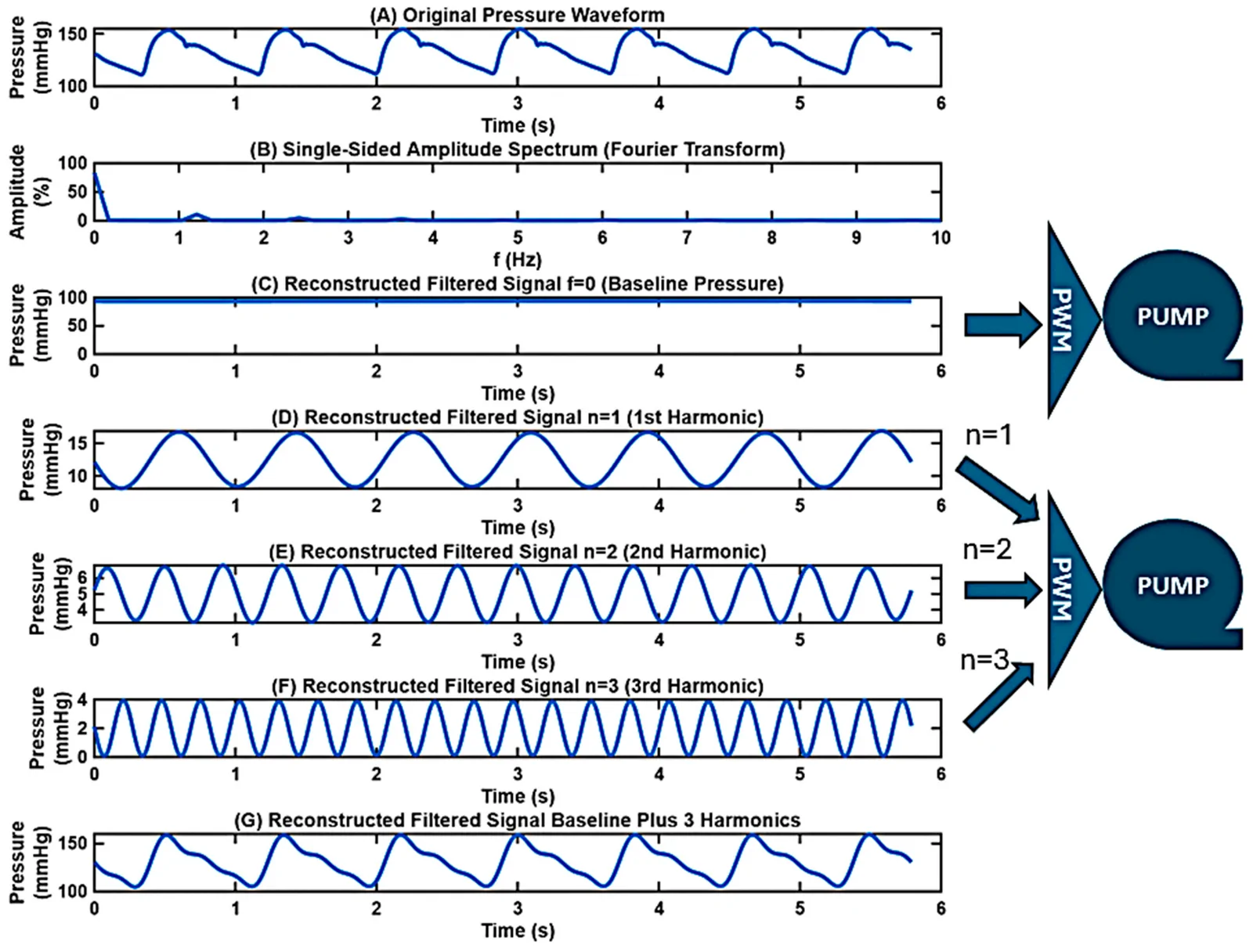
Fourier decomposition and waveform reconstruction of a representative brachial artery pressure waveform using MATLAB. From top to bottom: (**A**) the original arterial pressure waveform in the time domain; (**B**) the corresponding frequency-domain spectrum obtained using the Fast Fourier Transform (FFT); (**C**) the baseline (DC) component (*n* = 0) used to generate the baseline pump control signal; (**D**–**F**) the first (*n* = 1), second (*n* = 2), and third (*n* = 3) harmonic components, respectively, whose weighted amplitudes were combined to generate the waveform-shaping pump control signal; and (**G**) the reconstructed arterial pressure waveform obtained by combining the baseline component with the first three harmonics. The baseline and harmonic control components were converted to PWM signals for dual-pump actuation.

**Figure 2:**
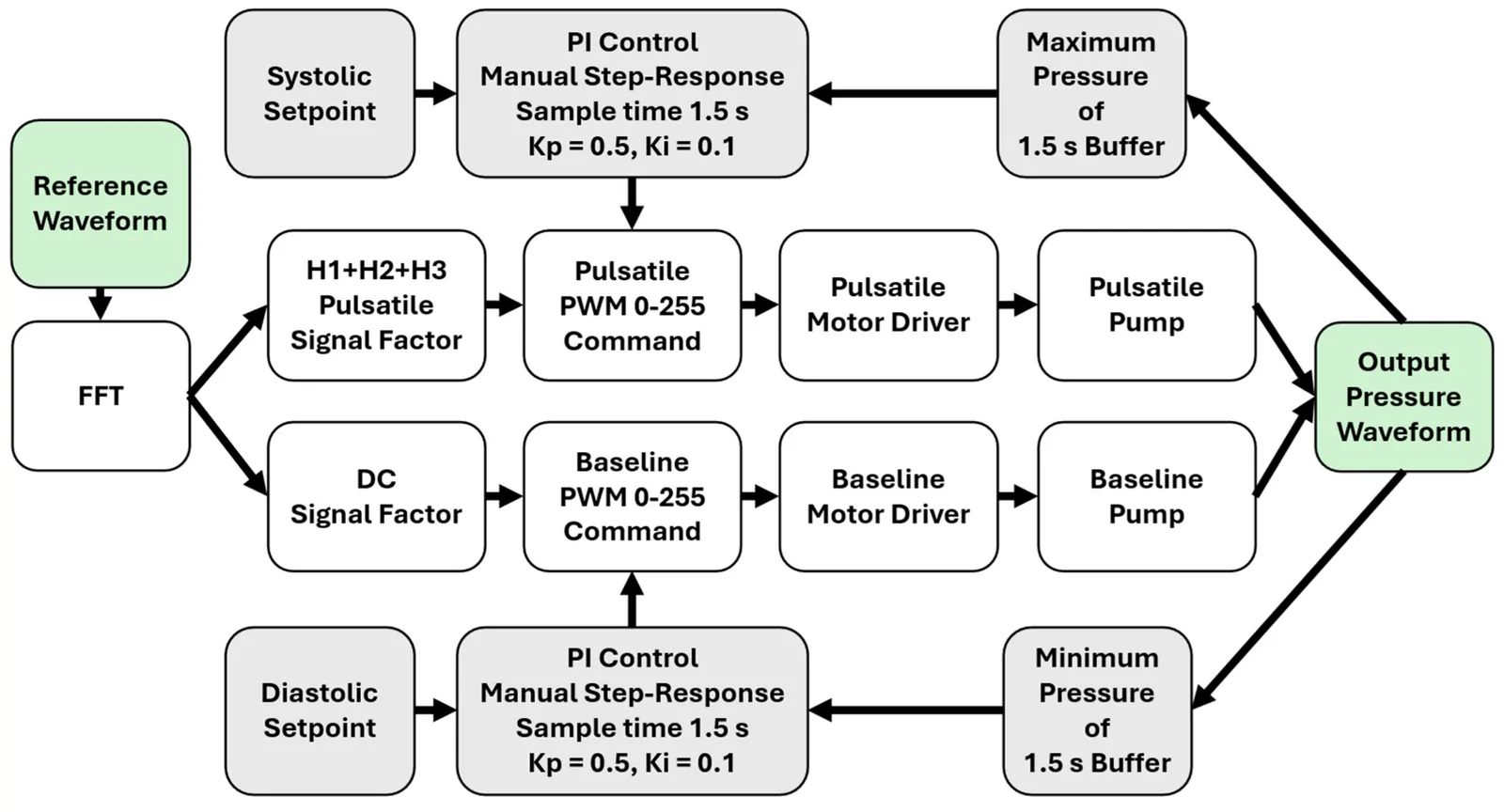
Control architecture of the proposed hMCL for reference arterial pressure waveform reproduction. The reference waveform is decomposed by FFT into DC and H1–H3 pulsatile components, which independently control the baseline and pulsatile pumps, respectively. Systolic and diastolic pressures are regulated using feedback-based proportional–integral (PI) controllers. The controller parameters, 1.5 s buffer interval, and PWM output range (0–255) are indicated.

**Figure 3:**
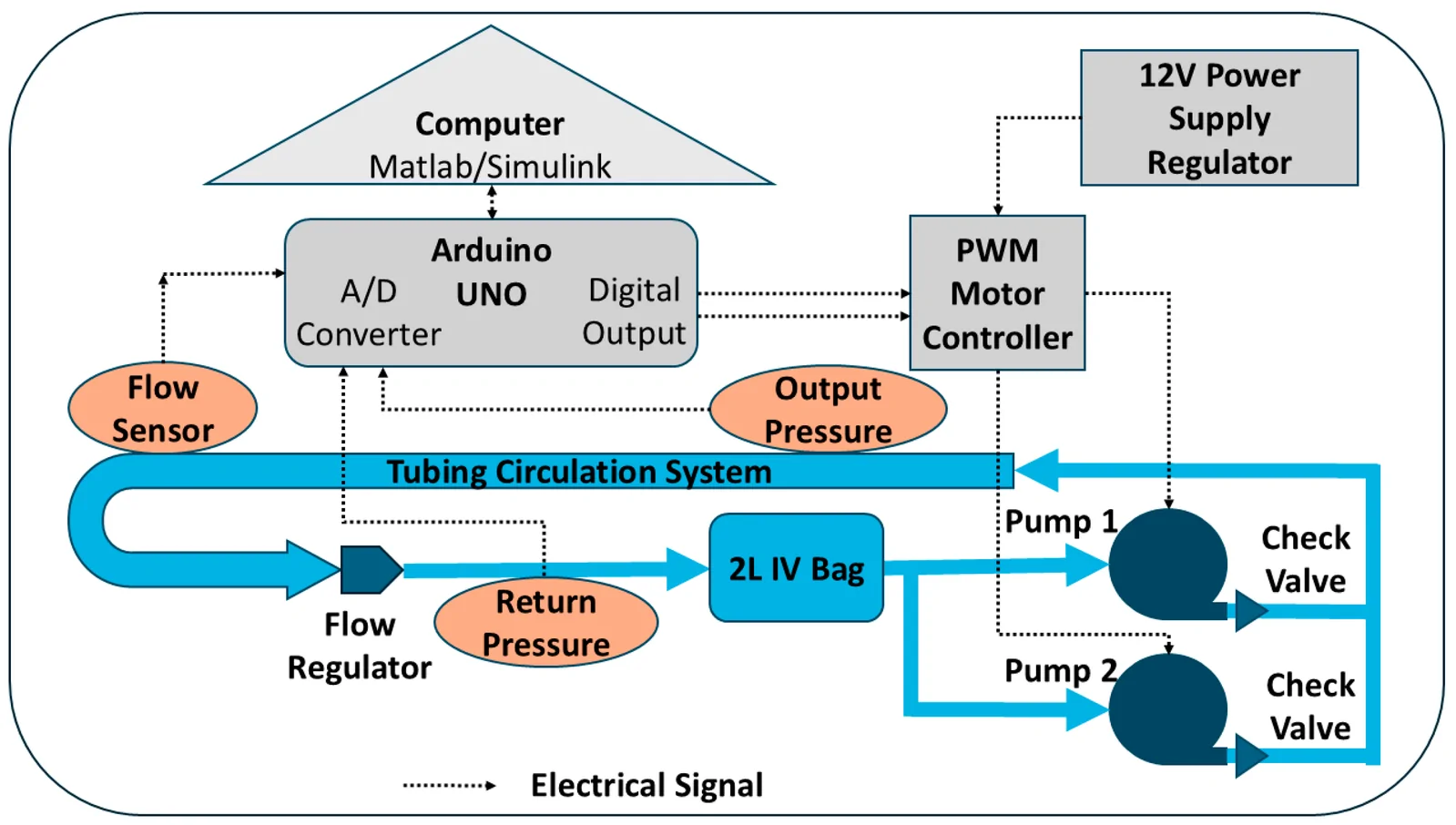
Schematic of the proposed dual-pump hMCL. MATLAB/Simulink communicates with an Arduino UNO microcontroller to independently regulate the baseline and pulsatile centrifugal pumps through PWM motor control. Pressure measurements provide feedback for closed-loop regulation of the hydraulic circulation system and arterial pressure waveform generation. Flow was monitored but was not independently validated as a waveform reproduction parameter in this study.

**Figure 4:**
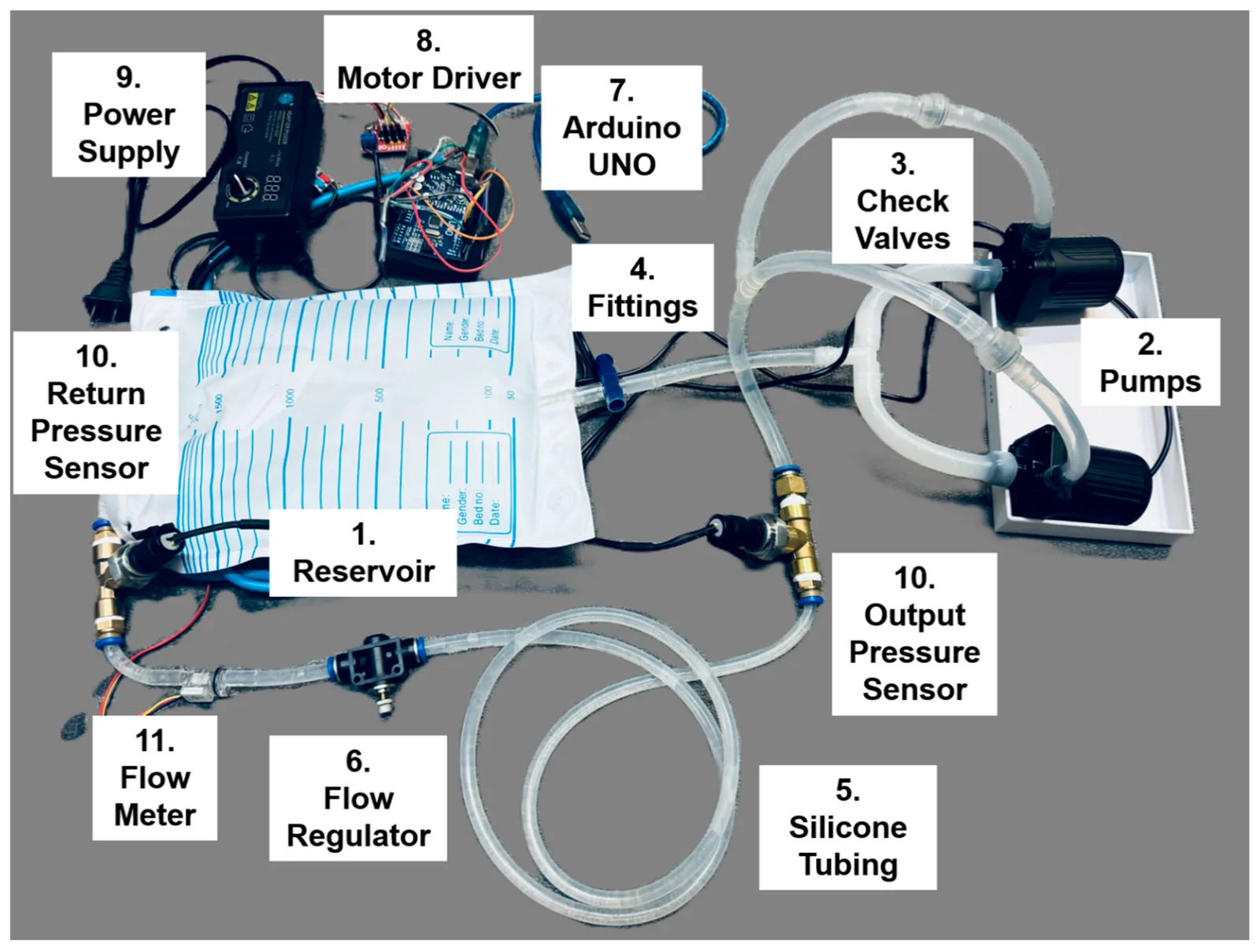
Photograph of the assembled dual-pump hMCL system. The system consists of (1) a reservoir, (2) two centrifugal pumps, each equipped with (3) a check valve to maintain unidirectional flow. The flow then passes through (4) silicone tubing and connectors into the (5) 1 m long silicone tubing that represents the systemic circulation. A (6) flow regulator adjusts the return flow to the reservoir, completing the closed-loop circuit. System operation is controlled by an (7) Arduino microcontroller, which interfaces with the (8) pump driver and (9) power supply to regulate pump speed. Two pressure sensors, the (10) outlet pressure sensor and return pressure sensor, continuously monitor system pressure and provide real-time feedback for closed-loop control. An (11) inline flow meter, positioned near the end of the circuit, measures the system’s volumetric flow rate.

**Figure 5:**
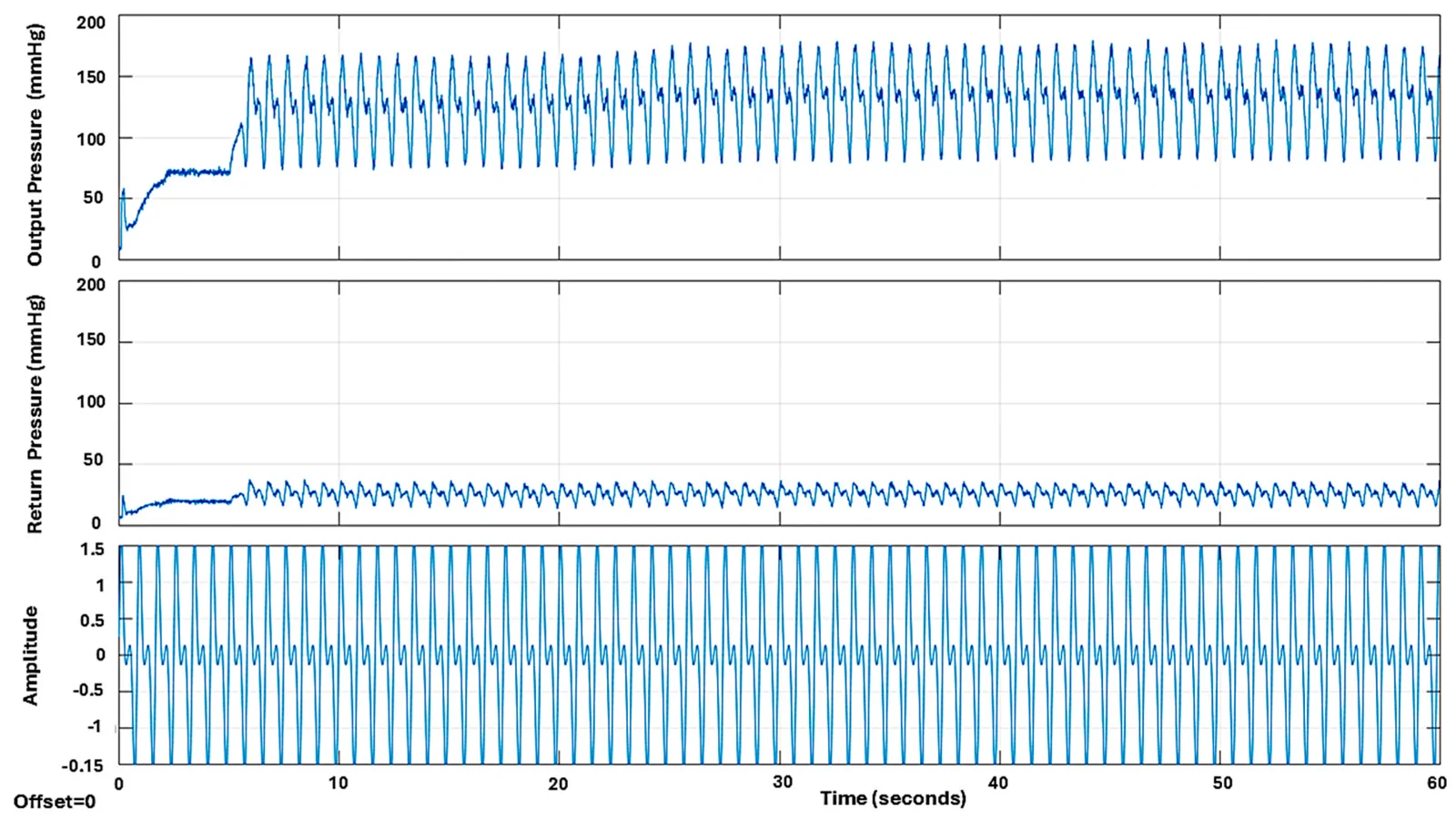
Representative Simulink screen capture during hMCL operation. Top: Generated arterial output pressure acquired from the pressure sensor and used for subsequent waveform analysis and validation. Middle: Return pressure monitored to verify stable hydraulic circulation and system operation during continuous testing. Bottom: Fourier-reconstructed reference waveform used by the Simulink controller to generate the dual-pump PWM control signal amplitude without offset. Following a brief startup transient, the system achieved stable periodic operation throughout one-minute acquisition period.

**Figure 6:**
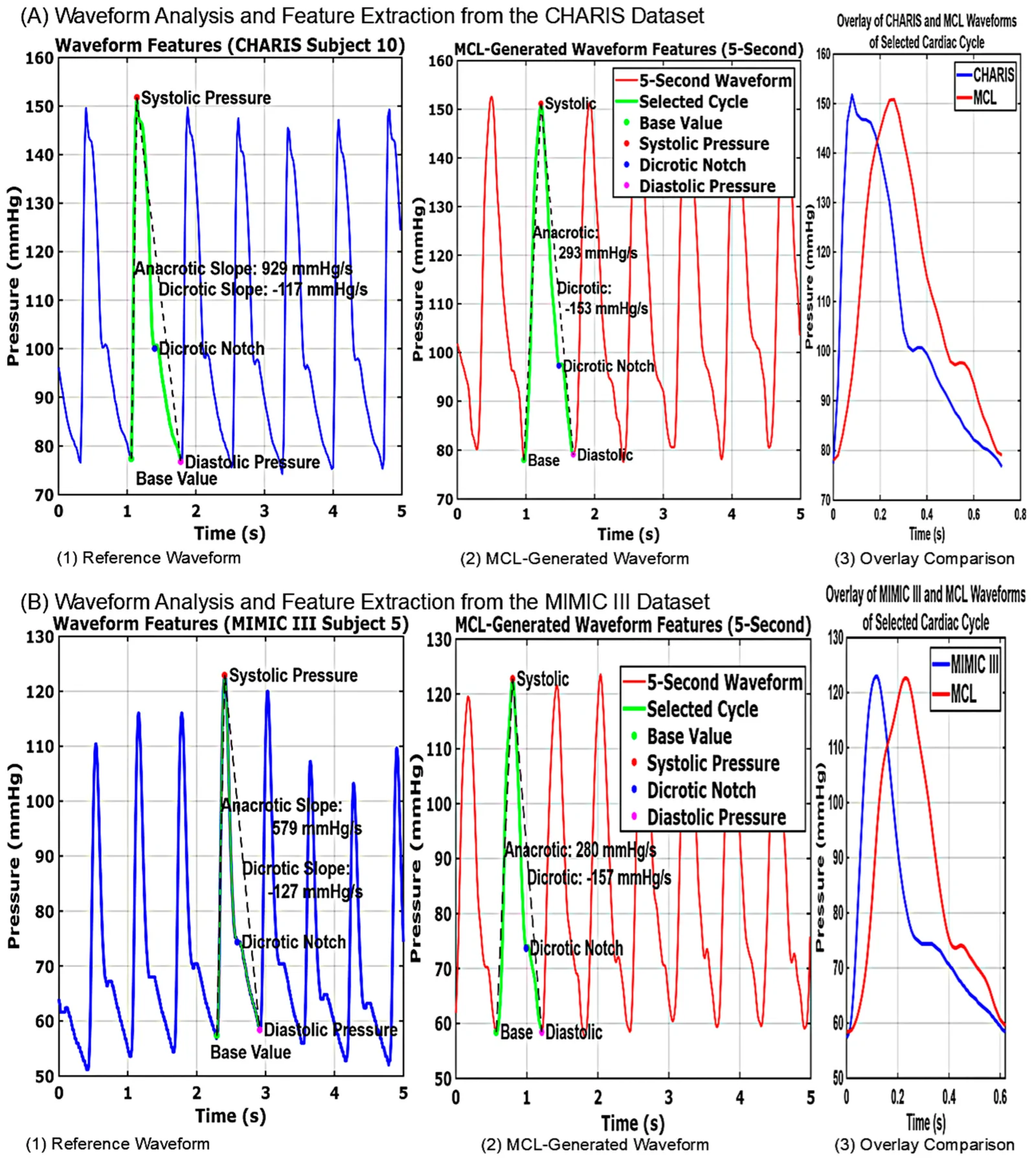

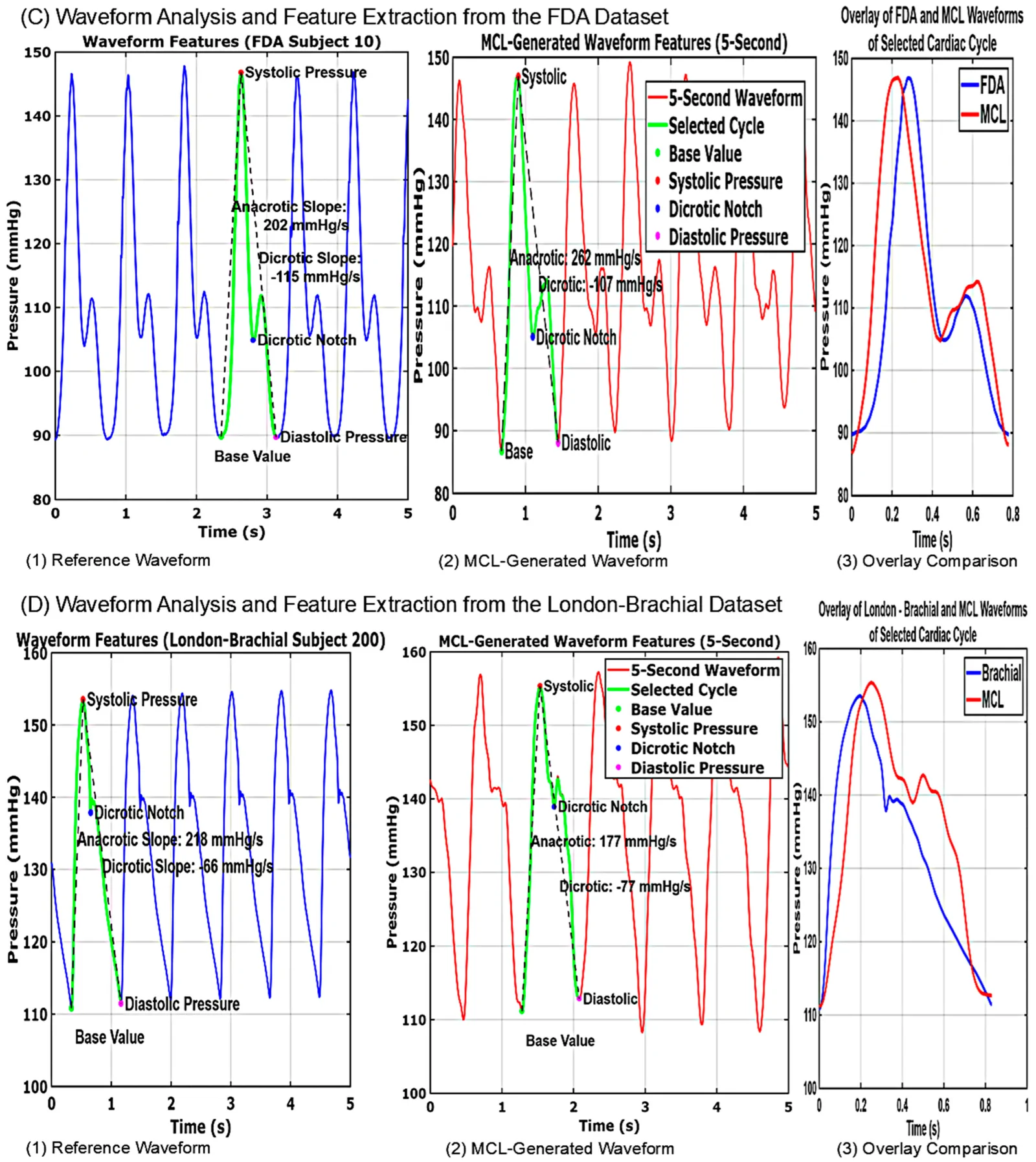

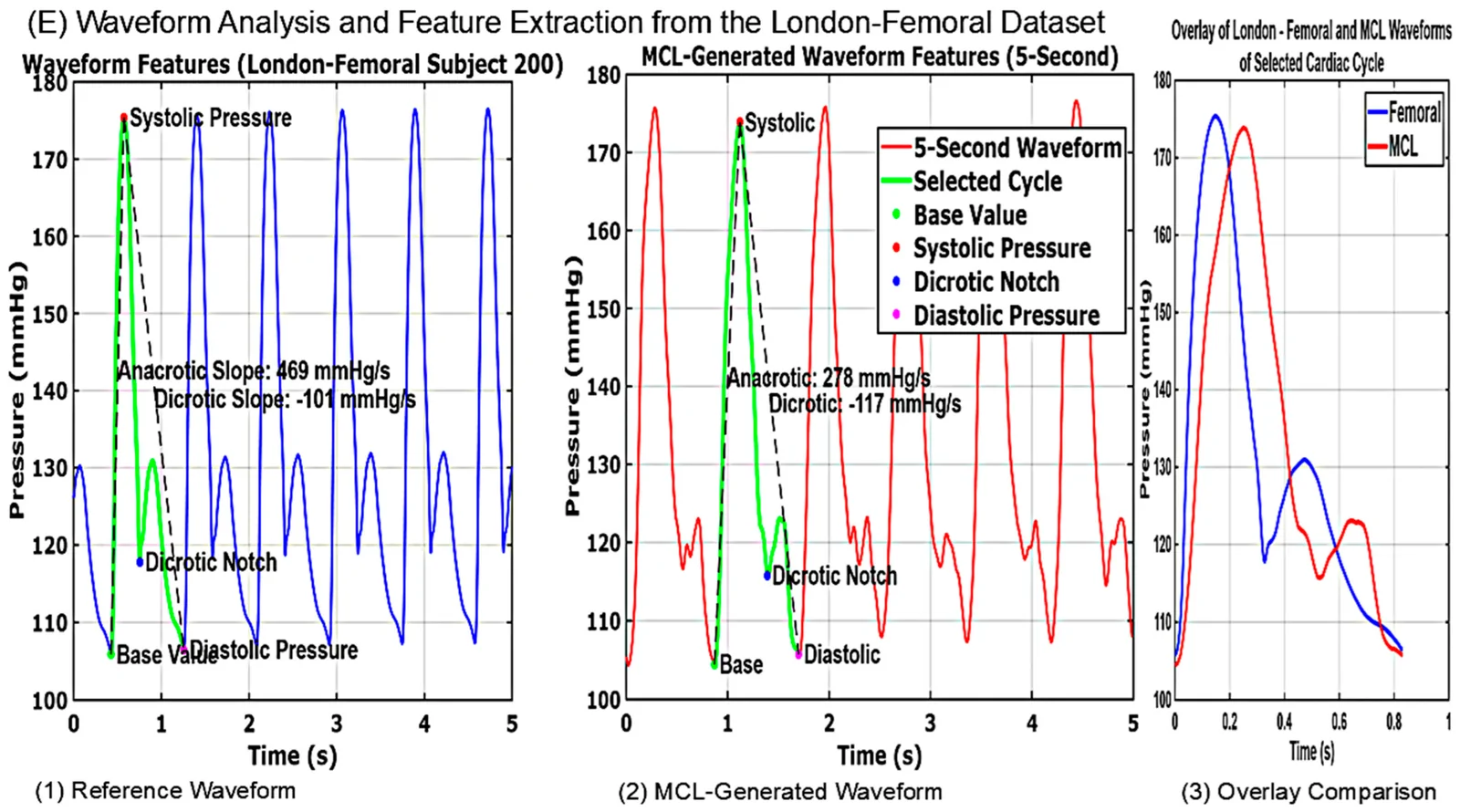
Waveform analysis and feature extraction for five representative reference arterial pressure waveform datasets: (**A**) CHARIS, (**B**) MIMIC III, (**C**) FDA MCL, (**D**) King’s College London—Brachial, and (**E**) King’s College London—Femoral. Within each dataset, (1) shows the reference arterial pressure waveform with the selected cardiac cycle and extracted fiducial features, (2) shows the corresponding MCL-generated waveform and extracted features, and (3) shows the overlay comparison of the selected cardiac cycles from the reference (blue) and MCL-generated (red) waveforms.

**Figure 7:**
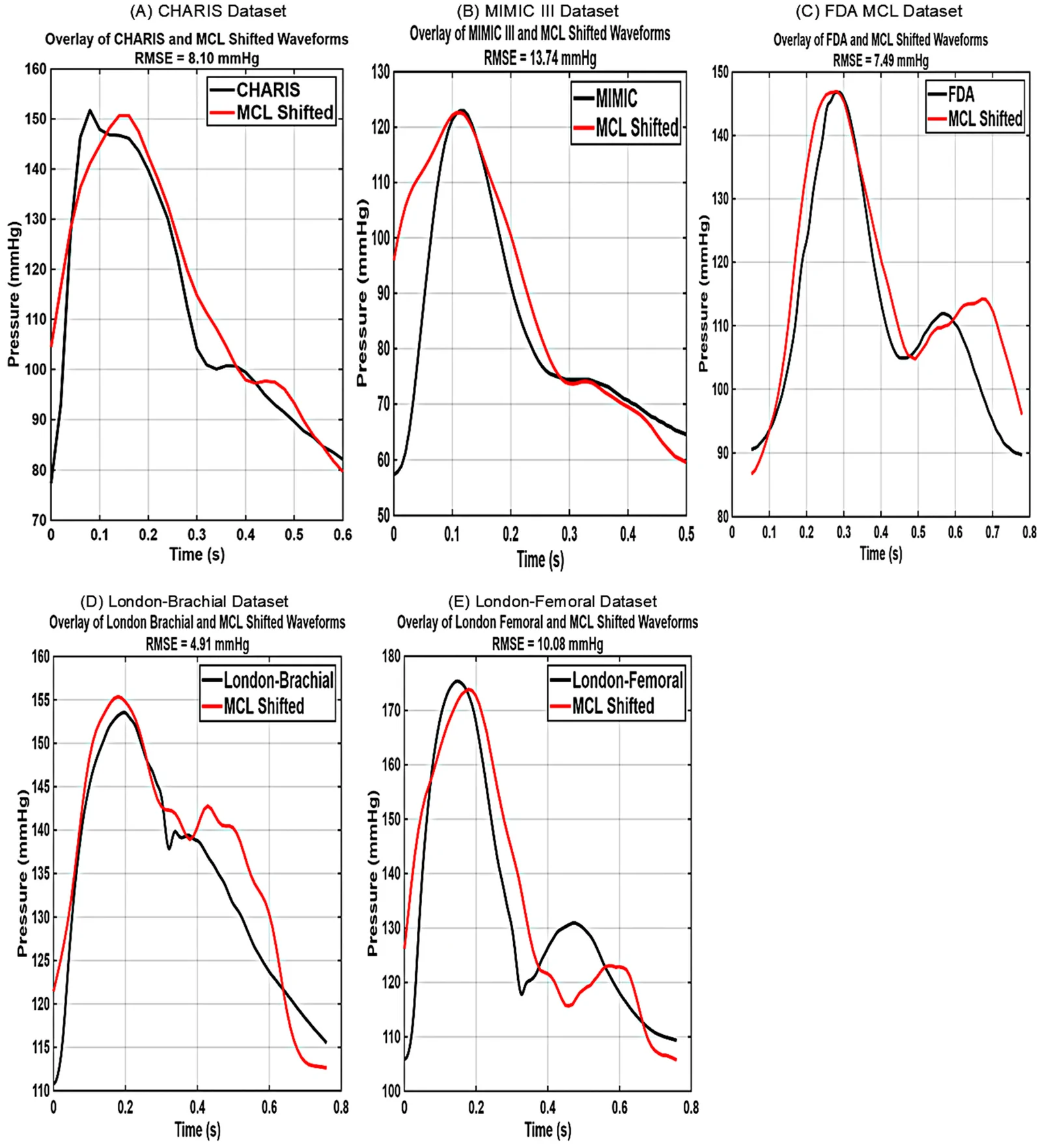
The overlay of the reference and time-shifted MCL waveforms for five representative datasets: (**A**) CHARIS, (**B**) MIMIC III, (**C**) FDA MCL, (**D**) King’s College London—Brachial, and (**E**) King’s College London—Femoral, demonstrating better RMSE.

**Figure 8:**
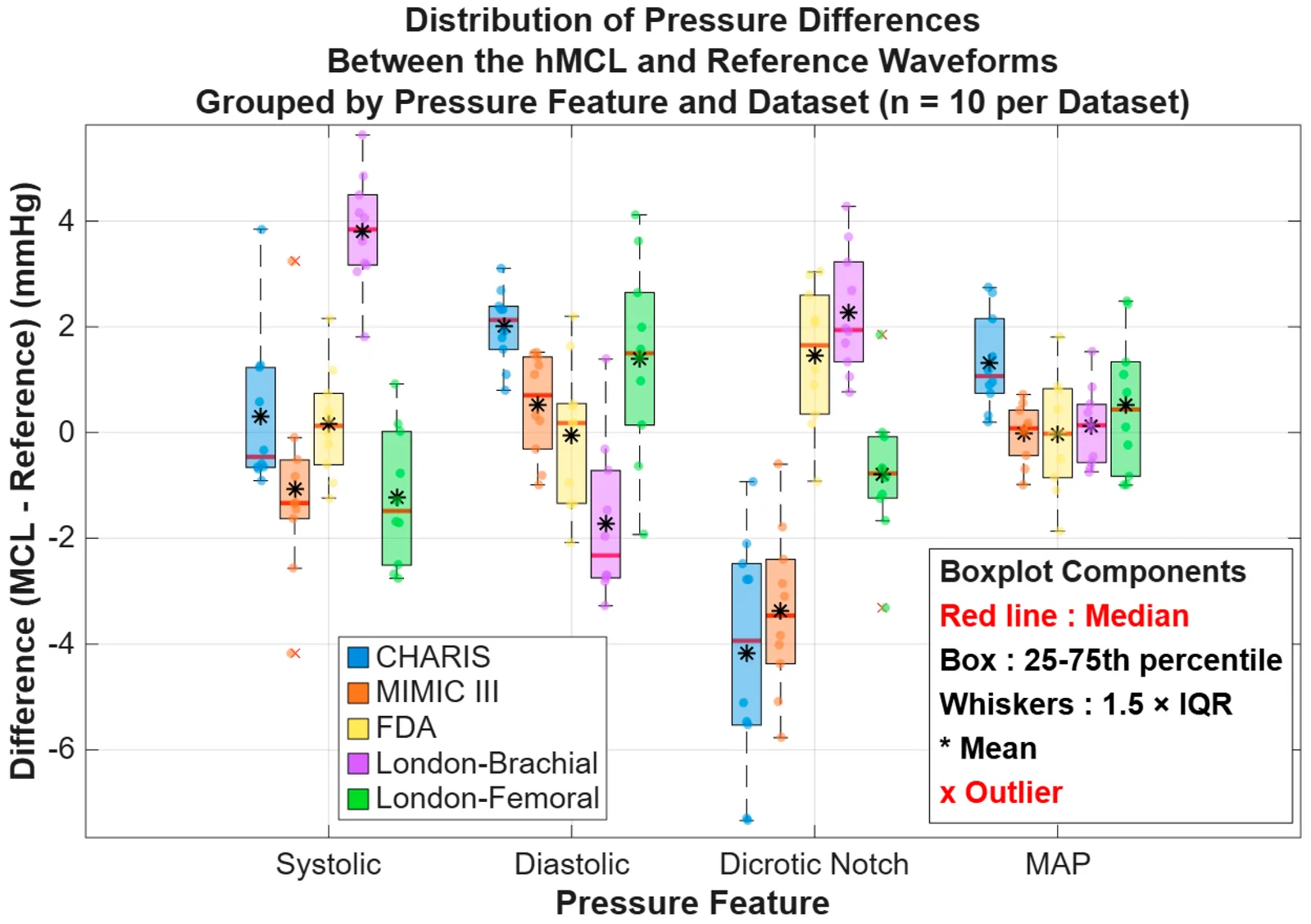
Grouped boxplot analysis of pressure feature differences between the hMCL and five reference waveform datasets: CHARIS, MIMIC III, FDA MCL, King’s College London—Brachial, and King’s College London—Femoral. Each marker represents the pressure feature difference obtained from an individual cardiac cycle. Box and marker colors identify the corresponding reference dataset. The red solid horizontal line indicates the median value, and the black asterisk (*) indicates the mean value. Dashed lines represent the full data range of the measured feature differences. Whiskers represent 1.5× interquartile range (IQR), and individual red crosses (X) indicate outliers.

**Figure 9:**
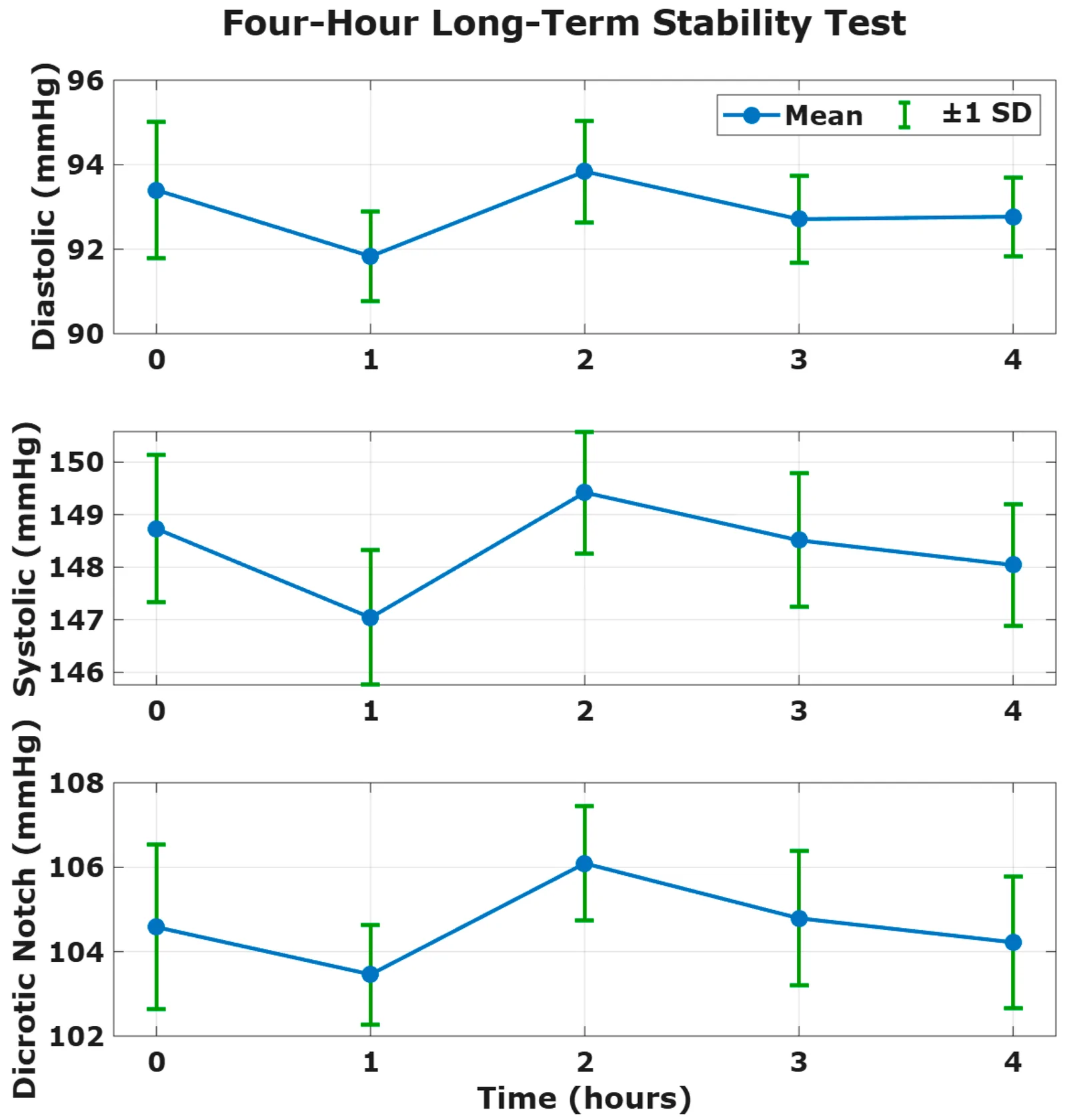
Long-term stability measurements over four hours. Three panels show (**top**) diastolic pressure, (**middle**) systolic pressure, and (**bottom**) dicrotic notch pressure. Data are presented as mean±SD at 0, 1, 2, 3, and 4 h.

**Table 1:** Fast Fourier Transform (FFT) analysis of five reference arterial waveform datasets, showing the fundamental frequency, normalized baseline (DC) amplitude, and normalized amplitudes (normalized to the full-scale waveform, 0–100%) and phase shifts (radians) relative to the fundamental harmonic for the first through fourth harmonics, the cumulative spectral power percentage of first through third harmonics, and the reconstruction RMSE using the first three harmonics (H1–H3) versus the first four harmonics (H1–H4).

Dataset	Fundamental Frequency (Hz)	Baseline (DC) Amplitude (%)	Amplitude (%) and Phase Shifts Relative to Fundamental Harmonic (Rad)				Cumulative Spectral Power	RMSE (mmHg)
			1st Harmonic	2nd Harmonic	3rd Harmonic	4th Harmonic	H1 + H2 + H3	H1 + H2 + H3 + H4
(1) CHARIS	1.40	**67.3**	**14.9** 0	**3.8** 1.4401	**0.8** 1.2326	**0.8** −3.3325	99.7%	7.90 7.68
(2) MIMIC III	1.60	**57.6**	**13.9** 0	**9.6** −0.0150	**5.1** −0.1806	**1.9** 0.0882	98.8%	5.22 5.13
(3) FDA MCL	1.28	**74.6**	**12.2** 0	**6.1** 2.9637	**3.9** 1.5870	**0.5** 2.8520	99.9%	3.71 3.55
(4) London Brachial	1.20	**86.6**	**10.9** 0	**4.5** −3.0750	**2.4** −0.1542	**0.7** −2.4428	99.6%	3.32 3.15
(5) London Femoral	1.20	**75.4**	**12.7** 0	**10.5** −3.9140	**4.7** −2.2452	**1.3** 1.0653	99.5%	5.33 5.03

Values in bold represent amplitude (%).

**Table 2:** Bill of materials and the cost of the dual-pump hMCL.

Item	Description	Cost (USD)
1	Reservoir: 2-Liter Urine Drainage (PVC) Bag	$2
2	(2) Centrifugal Pumps 12 Vdc 10 W	$30
3	(2) Plastic Check Valves 1/2″ Barb Hose	$6
4	Various Plastic Y-connectors and Fittings	$20
5	10′ Silicone 1/2″ (wall thickness 1/8″) tubing for main circulation and various sections of Silicone 3/8″ (wall thickness 1/16″) for connection	$20
6	Flow Regulator Needle Valve 1/4″ FNPT	$8
7	Arduino Uno R4 WiFi	$20
8	L298N Motor Driver Board Dual H-Bridge for DC Motor	$15
9	Power Supply Variable 3–12 V Regulator 60 W	$20
10	(2) Pressure Transducers, Stainless Steel 1/4″ MNPT 10 psi 5 V input, 0.5–4.5 V output (FUSCH G1/4)	$45
11	Flow Meter 1/4″ MNPT Hall Effect DC5V	$12
	Total	~$200 *

* Hardware cost was approximately US$200, excluding MATLAB/Simulink licensing and computer.

**Table 3:** Quantitative waveform reproduction error analysis across five reference arterial waveform datasets. RMSE values are reported before and after temporal alignment (time-shifted), with the latter representing the minimum achievable RMSE and the corresponding time shift.

Dataset	MAP Error	RMSE (mmHg)	Time Shifted (s)	Feature Errors		
				Systolic	Diastolic	Dicrotic Notch
(1) CHARIS	1.3%	24.1 *–8.1 ** mmHg	−0.101	0.4%	0.8%	2.8%
(2) MIMIC III	0.0%	22.1–13.7 mmHg	−0.120	0.1%	0.1%	0.9%
(3). FDA MCL	0.5%	12.3–7.5 mmHg	0.052	0.1%	1.8%	0.1%
(4) London Brachial	1.2%	9.1–4.9 mmHg	−0.054	1.2%	0.3%	0.8%
(5) London Femoral	0.8%	17.4–10.1 mmHg	−0.070	1.0%	0.6%	1.4%

* Result with MCL waveform synchronized with the reference waveform. ** Result with MCL waveform time-shifted to minimize the error.

**Table 4:** Descriptive statistical analysis of pressure differences between hMCL-generated and reference arterial waveforms across five datasets. Mean difference ± standard deviation (SD), median, interquartile range (IQR), 95% CI, and 95% LoA are reported for systolic pressure, diastolic pressure, dicrotic notch pressure, and MAP.

Dataset	Systolic (mmHg)					Diastolic (mmHg)					Dicrotic Notch (mmHg)					MAP (mmHg)				
	Mean ±SD	Median	IQR	95% CI	95% LoA	Mean ±SD	Median	IQR	95% CI	95% LoA	Mean ±SD	Median	IQR	95%CI	95% LoA	Mean ±SD	Median	IQR	95% CI	95% LoA
CHARIS	0.31 ±1.49	−0.46	1.89	−0.76, 1.38	−2.61, 3.23	2.00 ±0.71	2.13	0.82	1.50, 2.51	0.61, 3.39	−4.18 ±2.25	−3.94	3.05	−5.79, 2.57	−8.59, 0.23	1.33 ±0.91	1.07	1.41	0.68, 1.98	−0.45, 3.11
MIMIC III	−1.07 ±1.90	−1.34	1.11	−2.43, 0.29	−4.79, 2.65	0.53 ±0.97	0.71	1.74	−0.17, 1.22	−1.37, 2.43	−3.38 ±1.56	−3.47	1.97	−4.49, −2.27	−6.44, −0.32	−0.01 ±0.55	0.08	0.86	−0.40, 0.39	−1.09, 1.07
FDA	0.17 ±1.02	0.13	1.35	−0.56, 0.90	−1.83, 2.17	−0.05 ±1.37	0.18	1.89	−1.03, 0.93	−2.74, 2.64	1.46 ±1.33	1.64	2.25	0.50, 2.41	−1.15, 4.07	−0.04 ±1.08	−0.02	1.69	−0.81, 0.74	−2.16, 2.08
London—Brachial	3.81 ±1.08	3.84	1.33	3.03, 4.58	1.69, 5.93	−1.73 ±1.47	−2.33	2.03	−2.78, −0.68	−4.61, 1.15	2.27 ±1.17	1.94	1.89	1.43, 3.10	−0.02, 4.56	0.11 ±0.74	0.14	1.10	−0.42, 0.65	−1.34, 1.56
London—Femoral	−1.23 ±1.28	−1.48	2.53	−2.15, −0.31	−3.74, 1.28	1.39 ±1.86	1.50	2.51	0.06, 2.73	−2.26, 5.04	−0.78 ±1.32	−0.77	1.16	−1.73, 0.16	−3.37, 1.81	0.52 ±1.32	0.44	2.17	−0.42, 1.46	−2.07, 3.11
Overall	0.40 ±2.27	−0.16	3.05	−0.25, 1.04	−4.05, 4.85	0.43 ±1.83	0.67	2.57	−0.09, 0.95	−3.16, 4.02	−0.92 ±2.98	−0.77	4.56	−1.77, −0.08	−6.76, 4.92	0.38 ±1.05	0.26	1.35	0.08, 0.68	−1.68, 2.44

**Table 5:** Summary of waveform reproduction errors and statistical analysis of five independent trials.

MCL Run	Feature Errors			Feature Mean and Standard Deviation (mmHg)		
	Systolic	Diastolic	Dicrotic Notch	Systolic	Diastolic	Dicrotic Notch
1	0.51%	0.74%	0.05%	146.35 ± 1.14	87.53 ± 1.80	105.48 ± 2.29
2	0.21%	0.55%	2.00%	146.57 ± 1.10	87.87 ± 2.00	106.04 ± 2.57
3	0.09%	2.05%	1.03%	146.64 ± 1.00	87.21 ± 1.93	105.65 ± 2.32
4	0.72%	1.35%	1.73%	146.71 ± 1.16	87.37 ± 1.80	105.35 ± 2.91
5	0.62%	2.17%	2.74%	146.88 ± 1.07	87.00 ± 1.83	105.81 ± 2.62
Mean	0.43%	1.37%	1.51%	CV * = 0.75%	CV * = 2.14%	CV * = 2.41%

* CV, coefficient of variation.

**Table 6:** Comparison of representative conventional MCL systems. Qualitative ratings are based on the characteristics reported in the cited references and are intended to facilitate comparison of system capabilities rather than provide standardized performance rankings.

System	Pump Configuration	Waveform Generation	Programmability *	Approximate Cost **	Pressure Waveform Fidelity ***	Fourier-Based Harmonic Synthesis
Pantalos et al. (2004) [6]	Pneumatic chamber	Windkessel compliance and resistance tuning	Limited	High	High	No
Schampaert et al. (2014) [20]	Mechanical hydraulic loop	Pneumatic pressure regulator and compliance tuning	Limited	High	High (limited dicrotic notch reproduction)	No
Packy et al. (2022) [4]	ViVitro pulsatile pump	Multiple compliance chambers with electronically controlled pressure regulation	Moderate	High	High	No
Jeong et al. (2023) [3]	Pneumatic pump	Pneumatically controlled compliance chamber	High	Not reported	High	No
Commercial ViVitro SuperPump [27]	Pulsatile mechanical pump	Direct waveform programming	Moderate	>US$20,000	High	No
Proposed hMCL	Dual centrifugal pumps	Fourier harmonic synthesis with independent PWM control of systolic and diastolic pumps	High	<US$200	High (validated reproduction of systolic, diastolic, and identifiable dicrotic notch features)	Yes

* Qualitative assessment and classification criteria: Programmability was classified as high when waveform characteristics could be modified electronically with minimal system reconfiguration, moderate when electronic control was available but constrained by mechanical tuning and limited when waveform generation depended primarily on manual adjustment of compliance and resistance elements. ** Estimated hardware cost based on published information or commercially available components; excludes software licenses, instrumentation, labor, and custom fabrication. *** Qualitative assessment based on reported pressure waveform reproduction in the referenced publications. The proposed hMCL assessment is supported by the validation results presented in this study.

## Data Availability

The data presented in this study are derived from publicly available resources, including the PhysioNet CHARIS Database (DOI: 10.13026/C24G6F), the MIMIC-III Waveform Database (DOI: 10.13026/C2294B), the FDA Mock Circulatory Loop Database (DOI: 10.1016/j.compbiomed.2023.106979), and the King’s College London Haemodynamics Database (http://haemod.uk/original, accessed on 15 September 2026). A representative dataset sufficient to reproduce the principal findings of this study has been provided to the publisher and is available with the published article. Additional processed data, MATLAB/Simulink models, Arduino control code, and supporting materials are available from the corresponding author upon reasonable request.

## Abbreviations

The following abbreviations are used in this manuscript:

CI	Confidence Interval
CV	Coefficient of Variance
CVD	Cardiovascular Disease
DC	Direct Current
FDA	Food and Drug Administration
FFT	Fast Fourier Transform
hMCL	Hybrid Mock Circulatory Loop
IQR	Interquartile Range
LoA	Limits of Agreement
MAP	Mean Arterial Pressure
MCL	Mock Circulatory Loop
PWM	Pulse-Width Modulation
RMSE	Root Mean Square Error
SD	Standard Deviation
